# Pigeon and Poultry Breeders, Friends or Enemies of the Northern Goshawk *Accipiter gentilis*? A Long-Term Study of a Population in Central Poland

**DOI:** 10.3390/ani9040141

**Published:** 2019-04-02

**Authors:** Jakub Gryz, Dagny Krauze-Gryz

**Affiliations:** 1 Department of Forest Ecology, Forest Research Institute; Braci Leśnej 3, 05-090 Sękocin Stary, Poland; 2 Department of Forest Zoology and Wildlife Management, Faculty of Forestry; Warsaw University of Life Sciences, Nowoursynowska 159, 02-776 Warszawa, Poland; dagny.krauze@wl.sggw.pl

**Keywords:** field and forest mosaic, domestic pigeons and poultry, pellet analysis, population density, breeding parameters

## Abstract

**Simple Summary:**

The population of goshawks crashed in the middle of the 20th century due to persecution, forest management practices, and the usage of toxic pesticides (DDT) in agriculture. Now, it has rebuilt, yet the population trend is not equal across countries. Here, we focused on a goshawk population in central Poland for which monitoring started in the 1980s (high densities were recorded at that time of 16.3 pairs/100 km^2^) to see how changing environmental factors influenced the current population trend. In the field and forest mosaic, these birds build their nests in small forest complexes, but important prey tend to be caught near farmsteads. This has previously resulted in the persecution of the birds by farmers. Anthropogenic food (poultry and domestic pigeons) played a key role in their population density. Consequently, when the anthropogenic food base was limited (due to changes in the Polish farmland), population abundance dropped by half. As supplementary prey (including small-game and most corvid species) were not abundant, goshawks could not replace their staple food of anthropogenic origin. This demonstrates the complex way in which socioeconomic changes in agriculture can influence a raptor population: both positively (fewer cases of persecution are being recorded now) and negatively (small-scale breeding of pigeons and poultry became unimportant and unprofitable, and small game abundance decreased due to changes in farming practices and farmland structure).

**Abstract:**

In this study, we focused on a goshawk population in central Poland (study area 105 km^2^, forests 24 km^2^, seven small forest complexes) which was monitored long-term (with high densities recorded in the 1980s of 16.3 pairs/100 km^2^ despite persecution by farmers) to analyse how environmental factors (prey availability and changes in the forest structure) influenced population abundance, breeding parameters, and diet composition. The study was undertaken from 2011–2018, and the results were compared with published data from two previous study periods (1982–1992 and 2001–2003). The number of breeding pairs dropped from 17.1 to 8.0; the breeding success was around 75% in all study periods. The selection of nesting trees followed the changes in stand species and age structure. More nesting attempts per one nest were recorded in the current time period (1.7 vs. 1.1), which probably reflected lower anthropopressure (i.e., no cases of persecution were recorded in this study). Diet composition seemed to follow changes in the prey availability: The share of domestic pigeons and poultry (the main prey in the 1980s) as well as small game dropped, while the share of Eurasian jay and wood pigeon increased. Our studies suggested that anthropogenic food (poultry and domestic pigeons) played a key role for the goshawk population in the transformed habitats of the field and forest mosaic.

## 1. Introduction

The goshawk, *Accipiter gentilis,* is a widespread species inhabiting North America and Eurasia [1,2]. Its abundance in Europe fluctuated throughout the first half of the 20th century due to the persecution of raptors by hunters and forest management practices finally crashing in the 1950s and 1960s due to the usage of highly toxic pesticides (DDT) in agriculture [3,4,5,6,7]. Since the 1970s, the European population of goshawks started to rise moderately [3,8], yet this trend was not consistent for all countries. For example, in the UK, the goshawk went extinct in the 1980s. It was reintroduced, and its population grew in number; a systematic growth in the forest cover was conducive to this process of population restoration [5,9]. On the contrary, in Finland and Sweden, a decrease in goshawk abundance was recorded in the years 1980–2010 [10,11]. A population decrease was also noted in Poland in the last decade [12]. Similarly, in the USA, the population trend was equivocal [13,14], and many authors pointed to the potentially negative influence of forest management practices on goshawk populations, both in Europe and in North America [6,14,15,16]. 

Population densities in Poland in the last thirty years were between 0.5–10 pairs/100 km^2^, and generally, the highest numbers were found in areas with a high forest cover [17]. Yet, the record density in Poland, one of the highest in the world (16.3 pairs/100 km^2^), was recorded in the 1980s in the field-forest mosaic of central Poland (in the vicinity of Rogów village), an area with very low forest cover [18,19,20,21]. Goshawks in this area preyed mainly on domestic pigeons, *Columbia livia domestica*, and poultry, which together constituted over 70% of all their consumed biomass [19,22,23]. This high availability of anthropogenic food was probably the factor that allowed this population to reach such a high density [19]. On the other hand, the population was illegally persecuted, as birds were shot and trapped and their nests were cut down by local farmers [24,25]. Such practices took place across the whole of Poland (despite legal protection of goshawks since 1976; before this, raptors were treated as pests) [4,25], as in other countries [4,26,27,28]. In Poland, there is no reimbursement for damages done by raptors (on the contrary to, for instance, wolves (*Canis lupus*), lynxes (*Lynx lynx)*, and brown bears (*Ursus arctos*), while race pigeons (whose rings were often found in goshawk pellets) can be very valuable). 

This central Poland study area is where the goshawk population has been studied long term for almost 40 years [19,23,24,29,30,31,32] and where a population drop followed the record density found in the 1980s [24,30]. In our current study (2011–2018), we continued the long-term monitoring of the goshawk population in central Poland. We compared data obtained in the current study with analogical data obtained in the previous study periods [19,23,24,29,31]. Therefore, we were able to analyse crucial environmental factors (i.e., the main and complementary prey availability and changes in the forest structure) that may have influenced the population density of the goshawk, its breeding parameters, and its diet composition. 

## 2. Materials and Methods

The study is based on fieldwork done in the years 2011–2018, and the published data from the two previous study periods (i) 1982–1992 [19,23,29] and (ii) 2001–2003 [24,31] were used for comparison.

### 2.1. Study Area

**Figure 1:**
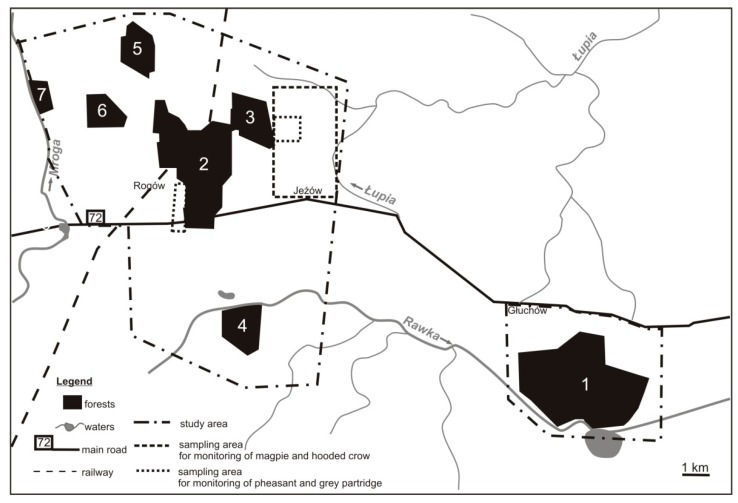
The distribution of forest complexes in the study area (central Poland, vicinity of Rogów village, Experimental Forest Station of Warsaw University of Life Sciences): Approximate borders of the whole study area for the Northern goshawk inventory and sampling areas for the monitoring of magpie, hooded crow, pheasant, and grey partridge are shown.

The studies were undertaken in a lowland area of central Poland in the area of the Experimental Forest Station of Warsaw University of Life Sciences in the vicinity of Rogów village (51° 49’ 17,98 N, 19° 53’ 54, 15 E). This region is affected by the mild oceanic climate of Western Europe and the harsh and dry continental climate of Eastern Europe and Asia. The duration of the growing season is approximately 210 days; the total precipitation is 600 mm per year; and the mean ambient temperature ranges from –4°C in January to +18°C in July. The study area comprised ca. 105 km^2^ of field and forest mosaic. Forests accounted for 25% of the area (approx. 2400 ha) and formed seven complexes (70–1000 ha) (Figure 1). The remaining area included arable lands (59%), orchards (5%), grasslands (5%), and scattered buildings [33]. Most of the forests (83% of their area) grew on rich, moderately moist soil. The main forest-forming species was Scots pine, *Pinus sylvestris*, but its share has dropped in the last decades as the species structure of stands has adjusted to habitat types. On the contrary, the share of larch *Larix* spp. and broadleaved species rose. Also, the share of older stands, which are best for goshawks to build their nests, has increased in recent decades (Table 1).

The mean stand age and stand volume has increased in recent decades, and in January 2009, it reached 71 years and 281 m^3^/ha, as compared to 51 years and 213 m^3^/ha in 1978. The annual harvest in the years 2014–2017 was rather low (4.9 m^3^/ha) [34]. 

### 2.2. Inventory of Goshawk Population and Measurements of Nesting Trees

Each year (2011–2018, January–March), all known nests were visited in all seven forest complexes. Courtship displays, as well as the presence of single birds, were noted. From the beginning of April to early May, newly built nests were searched by two people equipped with binoculars (10 × 42) and handheld transceivers, walking 50–200 m apart, depending on the stand type and age and visibility. All stands, except areas with newly planted trees and sapling stands, were searched. After the breeding season (in autumn), areas where nests might have been missed (i.e., where calls of adult or young birds were heard during the breeding season) were checked again. All nests that were located were recorded on the map of stands (1:10,000) and recorded on a GPS receiver (Garmin 62sc, Garmin 62sc, Garmin International, Inc., Olathe, KS).

All occupied nests were visited in June and July to estimate the number of fledglings. The reasons for brood loss were determined when possible. The criteria for breeding were similar to the previous periods (1982–1992 and 2001–2003) and were as follow: (i) mating behavior (calls and flights of two birds) in the vicinity of a new/rebuilt nest, (ii) incubating a female on a nest/remnants of goshawk eggs below the new/rebuilt nests, and (iii) the presence of chicks and fledglings on the/next to the nest or their remnants in the vicinity of a nesting tree. On this basis, we calculated the number of nesting attempts per one nest, i.e., the number of breeding cases divided by the number of nests used for breeding. The breeding success (percent of pairs that successfully produced at least one fledgling as referred to all breeding pairs) and the number of fledglings were calculated, both for successful pairs and for all known breeding pairs.

Measurements of the nesting trees were completed in November and December. The tree species were determined, and the height of the nest placement and the tree diameter at a 1.3 m height were measured. The altimeter SILVA Clino Master, Spencer Loggers Tape, and caliper Haglof Mantax Precision were used.

### 2.3. Diet Composition

Diet composition was assessed on the basis of an analysis of the plucking remains, pellets, and other prey remains and were collected on average every two weeks (from April to July) in the vicinity of nests (50 m radius). For each nest, we pooled all prey remains collected during the visits in one breeding season. In the laboratory, prey remains were systematically assigned with the aid of keys [35,36,37,38,39]. Furthermore, collections of feathers and skulls were used for comparison. In some cases, a histological analysis of the hair was performed [40]. Bird presence was detected mostly on the basis of feathers from plucking sites, while pellets were used mainly to detect other prey. We tried to avoid the double counting of prey detected with the two methods at one nest, i.e., we counted birds from pellets only in cases when we found bird remnants (mainly skulls) of species that were not detected on the basis of feathers from plucking sites. The mean prey body mass was adopted on the basis of the literature [35,41,42,43], and the body mass of small rodents was assessed during live-trapping undertaken every year in the same study area [44]. For bigger animals (assumingly eaten as carrion), the mean daily food requirement (200 g) was adopted as consumed biomass, while for insects, 1 g was adopted as a body mass for all species. The diet composition was presented as the share of a given taxon in a total number of prey items or consumed biomass. 

### 2.4. Food Base Assessment

The food base assessment included (i) anthropogenic food (domestic pigeons and poultry) as the main food component in the 1980s [23]; (ii) small game species (brown hare *Lepus europaeus*, European rabbit *Oryctolagus cuniculus*, pheasant *Phasianus colchicus*, and grey partridge *Perdix perdix*), which can be either a staple or supplementary prey [28,45,46,47,48]; and (iii) corvids (common magpie *Pica pica*, hooded crow *Corvus cornix*, rook *Corvus frugilegus*, and western jackdaw *Coloeus monedula*), which may serve as a supplementary food [27,49,50,51]. Whenever it was possible, we did the assessment in the same sampling areas and with the same method as it was done in the past study periods.

The availability of anthropogenic food (domestic pigeons, domestic ducks *Anas platyrhynchos domesticus*, domestic geese *Anser anser domesticus*, helmeted guineafowl *Numida meleagris*, and domestic rabbit *Oryctolagus cuniculus domesticus*) was not assessed for the two previous study periods. Now, it was assessed on the basis of door-to-door surveys supported by direct observations and along 22 transects (about 30 km in total) that run through villages. This was undertaken twice, in the summers of 2011 and 2018. All villages and farms that were located up to ca. 2 km from the forest complexes (Figure 1) were included. A minimal number of farms that “offered” anthropogenic food was counted. The farms where the animals were kept in enclosed areas (which means they were not available to goshawks) were not counted. 

In the first study period (1982–1992), a belt assessment was done to estimate the small game density [52,53]. With a low density, this method became inefficient, so spot light counts along transects intersecting open areas (mainly arable lands) were undertaken to assess the brown hare density (N/km^2^). They were done every year in December (2011–2017). The same route of ca. 40 km was repeated each year, and the detection distance was stable (200 m on one side). The counts were completed in favourable weather conditions (i.e., no rain, snow, or fog). Also, in the case of Galliforms, an alternative method was used. The counting of calling males (without playback) in spring was used to assess the density of grey partridges and pheasants. The first (2011–2013) section of work was undertaken in one sampling area of 1 km^2^, and in the years 2014–2018, a second sampling area (1 km^2^) was included (Figure 1). Both were located in open, arable land. The work was completed in the early mornings on three consecutive days in favourable weather conditions (i.e., no rain or wind). The maximum result from one of the three days was adopted as the final density (N ♂ km^2^) in a given year. Rabbit colonies were searched mainly in the field and forest ecotone, and in the fields, all earlier localities of colonies were checked; hunters, farmers, and employees of the Experimental Forest Station in Rogów were surveyed. 

In the case of magpies and hooded crows, occupied nests (in buffer strips, groups of trees/shrubs, or single trees) were searched every year in spring 2011–2018 in a sampling area of 1060 ha (arable land) located in the centre of the study area (Figure 1). The nests of rooks and western jackdaw were searched across the whole study area. The density of breeding pairs was obtained. Hooded crows, rooks, and jackdaws were studied in the first (1998–1992) and current study periods. For magpies, the same methodology as in the past [54] was applied.

### 2.5. Statistical Analysis

The normality of data distribution was checked with the Shapiro–Wilk test. To compare the abundance of breeding pairs in the three study periods, the Kruskal–Wallis test with the post hoc Mann Whitney test were used. The detailed data on the number of offspring were unavailable for the first period (1982–1992), so we only compared data for the second study period (2001–2003) with current data using the Mann–Whitney U-test. The percentage of trees of different species on which goshawks placed their nests and the diet composition in the three study periods were compared by means of a chi-square test, with a Bonferroni correction for multiple comparisons (*p* < 0.02). 

## 3. Results

### 3.1. Changes in the Population Density

**Figure 2:**
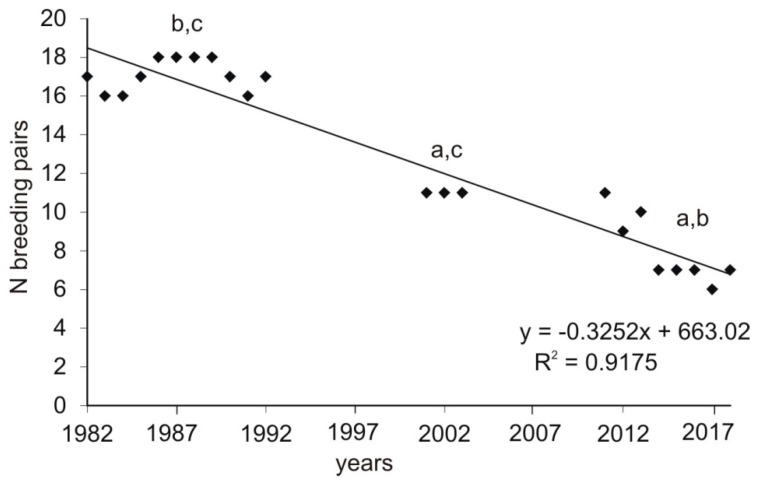
The number of breeding pairs recorded in the whole area of study (central Poland, vicinity of Rogów village, Experimental Forest Station of Warsaw University of Life Sciences) in the three compared periods: 1) 1982–1992 [19], 2) 2001–2003 [24], and 3) 2011–2018 (current study). The letters show the changes between the three study periods (a for 1982–1992, b for 2001–2003, and c for 2011–2018) as indicated by a Mann–Whitney post hoc test.

The current population densities (2011–2018) of goshawk were 0.76 pairs/10 km^2^ of total area and 3.3 pairs/10 km^2^ of forested area. Between 6 and 11 (mean 8.0, SD = 1.7) breeding pairs were recorded each year. The number of breeding pairs decreased when compared with the two previous study periods (Figure 2, Kruskall–Wallis test, H = 16.98, *p* < 0.0005; post hoc Mann–Whitney test, *p* < 0.05 for all comparisons between the three study periods). This means that, in comparison to the first study period (1982–1992), the population has decreased by almost 50%. Such a decrease in the number of breeding pairs was recorded in all forest complexes (Table 2).

### 3.2. Breeding Parameters

In the years 2011–2018, goshawks produced on average 1.6 fledglings per breeding pair (SD = 1.07) and 2.0 (SD = 0.69) per successful pair. The breeding success was 76% (SD = 7.2). Compared to previous study periods, these parameters seemed to be similar or decrease (Table 3). However, it was only possible to compare the number of juveniles between two periods (2001–2003 and 2011–2018; for 1982–1992, the detailed data were unavailable), for which no statistical differences were found (Mann–Whitney U-test, number of fledglings per breeding pair: Z = 0.87, *p* > 0.5; number of fledglings per successful pair: Z = 1.52, *p* > 0.05). 

### 3.3. Characteristics of Nesting Trees 

Currently (2011–2018), goshawks tend to build their nests on Scots pines most often (ca. 46%), and larch and back alder were also important nesting trees. As compared to previous study periods, the share of Scots pine as a nesting tree dropped (chi-square test, χ^2^ = 28.39, df = 2, *p* < 0.0001), while the share of larch (χ^2^ = 22.97, df = 2, *p* < 0.0001) and black alder (χ^2^ = 11.84, df = 2, *p* < 0.005, Table 4) rose. The current mean nesting tree age was 84 years (SD = 18.4), the tree trunk diameter was 48 cm (SD = 12.1), and the height of nest placement was 21 m (SD = 2.2, Table 4). In the years 2011–2018, goshawks built 37 nests and they bred 64 times, equaling to 1.7 breeding attempts per one nest. This value was lower for the first time period (1982–1992) at 1.1 breeding attempts per one nest (goshawks built 163 nests and bred 177 times).

### 3.4. Food Composition

In the current study period (2011–2018), goshawks preyed mainly on domestic pigeons and poultry, which accounted for 24.1% of their total prey items (N = 1065) and 49.2% of the biomass consumed (211,664 g in total). From among wild birds, wood pigeons *Columba palumbus* and Eurasian jays *Garrulus glandarius* were most important. Mammals accounted for 11.5% of their prey items and 3.7% of their biomass, including small prey and, in some cases, carrion. Amphibians, reptiles, and insects were found in very few pellets (Appendix A, Table A1).

As compared to the previous study periods, a decrease in the share of domestic pigeons (from 38% of all prey items in 1982–1992 to 22.8% now) was recorded (chi-square test, χ^2^ = 78.42, df = 2, *p* < 0.0001). The same was evident in the case of poultry, which, in the first period, constituted over 10% of all biomass, while recently, it was just 3.3%. The share of small game (brown hare, European rabbit, pheasant, and grey partridge) in the current study was the lowest from all three study periods (χ^2^ = 22.03, df = 2, *p* < 0.0001). On the contrary, the share of Eurasian jay (χ^2^ = 47.79, df = 2, *p* < 0.0001) and wood pigeon (χ^2^ = 46.26, df = 2, *p* < 0.0001) increased (Table 5).

### 3.5. Prey Availability

We were not able to obtain data regarding the pigeon and poultry availability from the previous decades. However, the number of farms that had domestic pigeons and poultry available for goshawks decreased even within the last study period between 2011 and 2018. The number of farms with domestic pigeons dropped by 34% (from 64 in 2011 to 42 in 2018), and those with poultry dropped by 33% (from 182 to 122 farms). The interviewed farmers confirmed that small-scale pigeon and poultry production has been limited in the last 30 years. Neither in 2011 nor in 2018 were domestic rabbits recorded as available to goshawks. If present, they were kept in cages; however farmers reported that most of them had ceased rabbit breeding by the beginning of the 1990s. 

A far as other food sources are concerned, the small game density was very low in 2011–2018 (Table A2) and dropped notably as compared to the 1980s. Brown hares and grey partridges became very rare, while European rabbits went extinct in the area (Table 6).

The abundance of magpie did not change over time (Table 6). Although there were no data to compare the density of other corvid species, it was low, while rooks were not present.

## 4. Discussion

Our study showed that the goshawk population decrease observed in central Poland in the last few years (2011–2018) was combined with a forced diet shift, as a response to changes in anthropogenic prey (poultry and domestic pigeons) availability. A high share of such food was typical for central Poland populations of the species in the 1980s; for instance, in Kampinos National Park, domestic pigeons and poultry accounted for 55% of prey items [58]. Studies done in natural forest complexes in northeastern Poland showed a much lower share of anthropogenic food or its absence; instead, birds preyed mainly on jays, thrushes *Turdus* spp., and woodpeckers [42,59]. However, the density of goshawks in such habitats was much lower (in the Białowieża Primeval Forest, it was 10.8 pairs/100 km^2^, [42]) than originally recorded in our study area in the 1980s [19]). Domestic pigeons were also an important food source for these birds in northwestern Spain (2008–2011), accounting for 17% of prey and 25% of the consumed biomass [60]. Similar in our case, when the availability of domestic pigeons dropped (by 71% between 1983 and 2012, [60]), a decrease in their share in the goshawk diet and an increase in the consumption of jays was recorded. However, even afterward this shift, the population density was very high at 15.8 nesting territories/100 km^2^ [21]. The goshawk diet can vary, although it is usually bird-dominated, with medium-sized birds such as thrushes, pigeons, and corvids (i.e., rooks and jays) playing an important role [27,49,50,51]. The regional variety can be recorded; for example, in the diet of goshawks from the Iberian Peninsula reptiles are frequently consumed [46,60]. Goshawks may prey on medium-sized mammals when they are abundant. In Gotland, a Baltic island, goshawks preyed mainly on rabbits, corvids, and thrushes, where rabbits were available in high density. With a low rabbit density, goshawks preyed on corvids, thrushes, and other birds, but the rabbit share was negligible. In the low rabbit abundance area, the density of goshawk was twice as low [61]. Similarly, in northeastern Spain, the food base for goshawks were European rabbits and red-legged partridge *Alectoris rufa*, while domestic pigeons accounted for only a small share of prey items. When the rabbit population crashed, goshawks preyed more intensively on the red-legged partridge [46]. In boreal forest, goshawks prey mainly on forest grouses and mountain hare *Lepus timidus* [28,47,62]. 

To our knowledge, only in our study area in the 1980s was such a high share of poultry recorded [23]. This was probably due to a specific situation in farming at this time. During the communist years in Poland (1945–1989), people suffered from a shortage of food, mainly meat. Central (state-governed) food production was insufficient, while individual farmers could not legally sell their products and instead were obliged to sell them to state purchase points. Meat (in any form) was hardly available in shops, and to buy it, special tokens were needed. Illegal meat trading was severely punished (in extreme cases with the threat of the death penalty). In this situation, people living on farmland (and to some extent in cities) kept poultry (mainly hens), rabbits, and pigeons as a source of meat (breeding of these species for personal use was not limited or controlled). The breeding of race or fancy pigeons was also a popular hobby. Most farms kept a flock of hens, while in each village, a few large pigeon lofts were present. Birds (during the daytime) wandered/flew around farmsteads unprotected from raptors. As a result, they became a staple food for goshawks in farmland areas [23,58]. The situation changed after 1989, when the communist regime collapsed and the food supply became unlimited. As a result, poultry or pigeon production became less important. Small-scale poultry farming was replaced by industrial chicken houses, with thousands of birds, yet these were completely unavailable to goshawks. Additionally, the bird flu outbreak in the years 2006–2007 resulted in strict procedures around domestic bird keeping, in which birds were required to stay in enclosures with no contact with wild birds. Moreover, in the last 30 years, many farms were abandoned (with people migrating to cities) as farmers often found employment elsewhere and some of the farmsteads are used as summer houses now. These factors all resulted in a sharp decrease in food availability for goshawks. 

Although in the previous study periods no monitoring of availability of anthropogenic food was conducted, small-scale poultry and pigeon farming was very popular and did not seem to decrease [19]. At the same time, goshawks were persecuted by farmers who perceived them as pests and tried to limit their population illegally (i.e., by cutting nesting trees, bird trapping, and shooting). For example, in Kampinos National Park near the study area in central Poland, up to 20% of nests were damaged each year [63]. Ornithologists claimed that this persecution was a severe threat which may lead to the extinction of local populations [4]. Nevertheless, in our study area, this could not have been very efficient given the high reproductive success of the population at that time [19]. The population decrease observed in 2001–2003 coincided with socioeconomic changes in the Polish farmlands (and the consequent decreased availability of anthropogenic food); however, a limited access to supplementary foods (i.e., small game) could also have played a role. As it was shown earlier, goshawks can adapt to prey availability changes by switching to alternative food sources [46,60,61]. In the case of our study area, however, the possibility of doing so was limited. In the last two decades a sharp increase in red fox *Vulpes vulpes* numbers was recorded in central Poland [64] and across the whole country [65]. This resulted in a crash of small game abundance, related also to agriculture intensification [30,56,66]. As a result, goshawks lost their potential alternative prey source (i.e., pheasant, grey partridge, brown hare, and European rabbit). In central Poland, there are no forest grouses (i.e., western capercaillie *Tetrao urogallus*, black grouse *Tetrao tetrix*, and hazel grouse *Tetrastes bonasia*) which contribute to a food base for goshawks in northern Europe [48,62,67]. As our study showed, the density of other alternative prey (i.e., most corvid species) was relatively low. Instead, goshawks preyed more heavily on jays and wood pigeons, whose abundance rose in the last decades across the whole of Poland [12]. It seems that farmers have now stopped persecuting goshawks, as we recorded no cases of nesting tree cutting, yet this has not counterbalanced the food base loss. 

Additionally, predators do not seem to be the reason for the goshawk population decrease, as occurred in northeastern USA [28]. Goshawks may be killed by martens (*Martes foina* and *M. martes*); however, their abundance in the study area was stable over the last 40 years [44], and we detected no such cases in the last study period (2011–2018). Another potential threat may be predation from the white-tailed eagle (*Haliaeetus albicilla*), two pairs of which nested in the study area [68], yet interactions between the two species were never recorded in our study. All identified reasons for the brood loss were connected to unfavourable weather conditions (nest falling down due to heavy winds or a heavy snowfall during below zero (°C) temperatures in April/May). According to some authors, the forest management practices may affect goshawk population negatively [6,15,16,69,70], yet others have shown that goshawks can successfully nest in managed forests [45], artificial intensive forest plantations [71], or even in urbanized areas [72]. However, the forests in our study area have undergone rather positive changes in the last 40 years: The forest cover has remained stable (or even increased slightly due to land abandonment and natural succession as a result of changes in farming practices after 1989) and increased in the mean age of stands as determined by the share of stands in the highest class ages. In the 2011–2018 period, a nesting tree was cut down by foresters only once, and it happened outside of the breeding season. In spring, the affected birds built a new nest 100 m further away and bred successfully. A factor that should be noted is that the number of breeding attempts per one nest was higher than in the 1980s, which indicates that disturbances from local farmers or foresters are probably lower now. 

The birds we studied appear to have adapted to changes in the forest structure. In the second half of the XIX and in the beginning of the XX century, mainly Scots pine was planted in rich forest habitats in the forests in our study area, as it was the most profitable species. In the last 40 years, a systematic decrease in the share of this species in stands as a result of stand reconstruction has been recorded [73]. As a result, goshawks built their nests on larches more frequently, while pines were chosen less frequently than in the 1980s. With the higher mean stand age birds could place their nests higher and on trees that are older and larger in diameter. As such, trees (especially larch) give a more solid base for the nest; this may be the other reason for a given nest being used for a longer period of time than in the 1980s.

The breeding parameters used in this study were similar to those obtained for farmland of western Poland (2001–2002), where the mean number of juveniles per breeding pair was 1.4 and per successful pair was 2.0 and the breeding success was 72% [20]. Similar results come from north Wisconsin in the USA; the mean number of young fledged per active nest was 1.6 and per successful nest was 2.1, and the breeding success was 73% [28]. In northeastern Poland in the Białowieża Primeval Forest, productivity was low at just 1.1 juveniles per breeding pair [74]. In the more transformed habitat of the Augustów Primeval Forest, the breeding parameters were much higher: 1.9 juveniles per breeding pair and 2.7 per successful pair [59]. Nevertheless, the highest productivity per successful pair was recorded in central Poland in Kampinos National Park, where the birds produced on average 3.3 juveniles [63], in a population feeding heavily on domestic pigeons [58]. 

## 5. Conclusions

Overall, our study showed that anthropogenic food (poultry and domestic pigeons) plays a key role for the goshawk population in the transformed habitats of the field and forest mosaic, even though the hunting birds were persecuted by local farmers. The birds built their nests in small forest complexes, but much of their prey was caught in open spaces in the vicinity of farmsteads. Consequently, what has led to the observed population decrease is the current limited anthropogenic food base, as a result of political and socioeconomic changes that have affected polish farmland. As medium-sized mammals and birds (i.e., small game and most corvid species) are not abundant (or absent, like forest grouses), they could not replace the staple food of anthropogenic origin when it was removed. This study showed the complex way in which socioeconomic changes in agriculture influence the raptor population, both positively (with fewer cases of persecution since the small scale production of poultry and pigeons is no longer important and widespread) and negatively (by influencing the food base directly; in this case, by limiting anthropogenic food source availability; and indirectly, by changes in farming practices that have led to a decrease in small-game prey availability). Even though farmers have now stopped persecuting goshawks, this has not counterbalanced the anthropogenic food base loss.

## Figures and Tables

**Table 1:** The changes in the structure of stands in the study area (central Poland, vicinity of Rogów village, Experimental Forest Station of Warsaw University of Life Sciences) in recent decades [34]: The table lists stands that are old enough to place nests by the Northern goshawk and the main trees species available in the current period.

Stand Characteristics	Year of Assessment		
	1989	1999	2009
	% of Area		
stands 60–100 years old	36.4	34.2	42.1
stands over 100 years old	1.3	6.0	7.9
Scots pine *Pinus sylvestris*	76.9	69.9	49.9
larch *Larix* spp.	4.1	6.1	6.7
oaks *Quercus* spp.	10.7	13.8	19.4
common beech *Fagus sylvatica*	1.0	3.0	9.7

**Table 2:** The average number of breeding pairs in the three compared periods in seven forest complexes and in the whole area of study (central Poland, vicinity of Rogów village, Experimental Forest Station of Warsaw University of Life Sciences): The numbers of the forest complexes are as indicated in Figure 1 (map of the study area). The data are for 1982–1992 [19], 2001–2003 [55], and 2011–2018, the current study. The SD values are given when the data were available.

Forest Complex	Study Periods		
	1982–1992	2001–2003	2011–2018 (SD)
1	6.7	2.9	3.6 (0.7)
2	3.8	2.7	1.6 (0.9)
3	1.8	2.0	0.4 (0.5)
4	1.1	1.0	0.9 (0.3)
5	1.5	2.0	1.0 (0.0)
6	1.1	0	0 (0.0)
7	0.8	0	0.5 (0.5)
whole area	17.1	11.0	8.0

**Table 3:** The breeding parameters of the goshawk population in the area of study (central Poland, vicinity of Rogów village, Experimental Forest Station of Warsaw University of Life Sciences) in the three compared breeding periods: The data are for 1982–1992 [29], 2001–2003 [24], and 2011–2018, the current study. The SD values are given when data were available.

Breeding Parameter	1982–1992	2001–2002 (SD)	2011–2018 (SD)
number of fledglings per pair	2.1	1.8 (1.19)	1.6 (1.07)
number of fledglings per successful pair	2.8	2.4 (0.53)	2.1 (0.69)
breeding success (%)	75	75	76 (7.2)

**Table 4:** The characteristics of the nesting trees of goshawk in the area of study (central Poland, vicinity of Rogów village, Experimental Forest Station of Warsaw University of Life Sciences) in the three compared breeding periods: The data are for 1982–1992 [29], 2001–2003 [55], and 2011–2018, the current study.

Nesting Trees/Nests Characteristics	Study Period		
	1982–1992	2001–2003	2011–2018
Tree species (%)			
*Pinus sylvestris*	84.7	60.0	45.9
*Larix decidua*	5.5	13.0	32.4
*Abies alba*	0.0	13.0	2.7
*Picea abies*	1.2	7.0	2.7
*Quercus* spp.	3.1	0.0	0.0
*Alnus glutinosa*	2.4	7.0	16.2
*Betula verrucosa*	3.1	0.0	0.0
Tree measurements			
diameter (cm)	42	44	48
age (years)	75	77	84
Nest placement			
height (m)	18	20	21
number of nesting trees	163	15	37

**Table 5:** The share of selected prey categories in the goshawk diet in the area of study (central Poland, vicinity of Rogów village, Experimental Forest Station of Warsaw University of Life Sciences) in the three compared breeding periods: Small game included pheasant, grey partridge, brown hare, and European rabbit. The data are for 1982–1992 [29], 2001–2002 [31], and 2011–2018, the current study. Nd: no data available.

Prey Category	1982–1990 (%)		2001–2002 (%)		2011–2018 (%)	
	Prey Items	Biomass	Prey Items	Biomass	Prey Items	Biomass
Domestic pigeon	38.0	nd	32.3	57.4	22.8	45.9
Poultry	2.9	10.1	2.2	5	1.3	3.3
Wood pigeon	2.9	nd	6.8	14.3	9.0	21.8
European jay	2.9	3.0	8.1	6.3	13.1	11.6
Small game	2.7	4.0	2.6	3.4	0.3	0.3
total N prey items/biomass (g)	1 513	nd	310	69 629	1 065	211 664

**Table 6:** The changes in prey available to goshawks in the area of study (central Poland, vicinity of Rogów village, Experimental Forest Station of Warsaw University of Life Sciences) in the three compared study periods: The sources of data are from 1982–1992, 2001–2003, published data (references in square brackets), and 2011–2018, the current data. nd: no data available.

Prey	1982–1992	2001–2003	2011–2018
brown hare	30 ind./km^2^ [56]	8–13 ind./km^2^ [56]	2.1 ind./km^2^
European rabbit	at least five big colonies [57]	last rabbits in 2007 [57]	no colonies recorded
grey partridge	21–33 ind./km^2^ (autumn) [52]	nd	0.1 males/km^2^ (spring)
pheasant	5.7–11.0 ind./km^2^ [53]	nd	2.3 males/km^2^
magpie	1.6 pairs/km^2^ [54]	nd	1.6 pairs/km^2^
rook	nd	nd	no nests recorded
hooded crow	nd	nd	0.15 pairs/km^2^
jackdaw	nd	nd	0.25 pairs/km^2^

## Appendix A

**Table A1:** A detailed diet composition of the goshawks in the area of study (central Poland, vicinity of Rogów village, Experimental Forest Station of Warsaw University of Life Sciences) in the years 2011–2018 based on an analysis of the remains from the plucking sites and pellets.

Prey	N	Body Mass	Total Biomass	Biomass	Prey Items
		g		%	
*Columba livia* f. *domestica*	243	400	97200	45.9	22.8
*Gallus domesticus*	13	500	6500	3.1	1.2
*Numidia meleagris*	1	500	500	0.2	0.1
**∑ Anthropogenic birds**	**257**		**104200**	**49.2**	**24.1**
*Columba palumbus*	97	475	46075	21.8	9.1
*Streptopelia decaocto*	10	225	2250	1.1	0.9
*Streptopelia turtur*	1	150	150	0.1	0.1
*Columba oenas*	1	250	250	0.1	0.1
*Phasianus colchicus*	2	500	1000	0.5	0.2
*Coturnix coturnix*	1	100	100	0.0	0.1
*Anas platyrhynchos*	1	500	500	0.2	0.1
*Accipiter nissus*	1	200	200	0.1	0.1
*Vanellus vanellus*	1	280	280	0.1	0.1
*Scolopax rusticola*	1	300	300	0.1	0.1
*Chroicocephalus ridibundus*	1	300	300	0.1	0.1
*Cuculus canorus*	1	120	120	0.1	0.1
*Asio otus*	1	250	250	0.1	0.1
*Strix aluco*	1	500	500	0.2	0.1
*Dendropcopos major*	29	70	2030	1.0	2.7
*Dendrocopos* spp.	9	70	630	0.3	0.8
*Driocopus martius*	2	300	600	0.3	0.2
*Motacilla alba*	2	24	48	0.0	0.2
*Turdus philomelos*	58	50	2900	1.4	5.4
*Turdus merula*	34	70	2380	1.1	3.2
*Turdus pilastris*	15	70	1050	0.5	1.4
*Turdus viscivorus*	7	80	560	0.3	0.7
*Turdus* spp.	40	65	2600	1.2	3.8
*Phylloscopus* spp.	2	8	16	0.0	0.2
*Ficedula* spp.	4	15	60	0.0	0.4
*Parus major*	5	18	90	0.0	0.5
*Paridae* spp.	4	20	80	0.0	0.4
*Sitta europea*	18	22	396	0.2	1.7
*Lanius* sp.	4	50	200	0.1	0.4
*Garrulus glandarius*	140	175	24500	11.6	13.1
*Pica pica*	3	200	600	0.3	0.3
*Oriolus oriolus*	1	73	73	0.0	0.1
*Sturnus vulgaris*	30	80	2400	1.1	2.8
*Passer* spp.	4	25	100	0.0	0.4
*Fringilla coelebs*	12	20	240	0.1	1.1
*Chloris chloris*	3	26	78	0.0	0.3
*Coccothraustes coccothraustes*	29	55	1595	0.8	2.7
*Loxia* sp.	4	35	140	0.1	0.4
*Emberiza citrinella*	4	30	120	0.1	0.4
Small bird unident.	27	25	675	0.3	2.5
Medium bird unident.	40	75	3000	1.4	3.8
**∑ Aves**	**907**		**203636**	**96.2**	**85.2**
*Talpa europea*	2	80	160	0.1	0.2
*Apodemus agrarius*	4	24	96	0.0	0.4
*Apodemus* spp.	9	27	243	0.1	0.8
*Rattus norvegicus*	3	200	600	0.3	0.3
*Myodes glareolus*	28	18	504	0.2	2.6
*Microtus* spp.	6	22	132	0.1	0.6
Rodentia unident.	45	20	900	0.4	4.2
*Sciurus vulgaris*	3	300	900	0.4	0.3
*Oryctolagus cuniculus* f. *dom.*	3	200	600	0.3	0.3
*Lepus europeus* juv.	1	100	100	0.0	0.1
*Felis catus*	9	200	1800	0.9	0.8
*Mustela* sp.	1	45	45	0.0	0.1
*Vulpes vulpes*	1	200	200	0.1	0.1
*Canis familiaris*	3	200	600	0.3	0.3
*Sus domestica*	3	200	600	0.3	0.3
*Capreolus capreolus*	2	200	400	0.2	0.2
**∑ Mammalia**	**123**		**7880**	**3.7**	**11.5**
*Rana* sp.	1	15	15	0.0	0.1
*Natrix natrix*	1	100	100	0.0	0.1
Insecta unident.	33	1	33	0.0	3.1
**Total**	**1065**		**211,664**	**100.0**	**100.0**

**Table A2:** The changes in the density of small game species in the area of study (central Poland, vicinity of Rogów village, Experimental Forest Station of Warsaw University of Life Sciences) in the current study period (2011–2018).

Species	2011	2012	2013	2014	2015	2016	2017	Mean
brown hare (ind./km^2^)	3	4	1	2	2	1	2	2.1
European rabbit	no colonies recorded							
grey partridge (n males/km^2^)	0	0	0	0.5	0	0.5	0	0.1
pheasant (n males/km^2^)	2	1	3	2	3	3	2	2.3
